# Supplementary material for: KinOrtho: a method for mapping human kinase orthologs across the tree of life and illuminating understudied kinases
Source: BMC Bioinformatics. 2021 Sep 18;22:446. doi: 10.1186/s12859-021-04358-3 (PMC8449880; doi:10.1186/s12859-021-04358-3)
Supplement: Supplementary file 1 — Additional file 1. Supplementary results, figures, and tables. [file 12859_2021_4358_MOESM1_ESM.pdf]

METHODOLOGY ARTICLE

KinOrtho: a method for mapping human kinase orthologs across the tree of life and illuminating understudied kinases

Liang-Chin Huang[1], Rahil Taujale[1], Nathan Gravel[2], Aarya Venkat[3], Wayland Yeung[1], Dominic P Byrne[4], Patrick A Eyers[4] and Natarajan Kannan[1,3*]

Supplementary Results and Discussion

Unique KinOrtho-defined human kinase orthologs

We mapped the comparison results into the human kinome tree to visualize the orthology detection trends across human kinases. We calculated an overlap ratio as the number of one-to-one human kinase orthologs identified by KinOrtho (overlapping set) and other state-of-the-art orthology inference methods (Figure S2). The average overlap ratio for each human kinase is shown in Figure S3, and kinases with the least overlaps are labeled. The orthologs of these ten human kinases, such as understudied kinases "Cyclin-dependent kinase 19" (CDK19; synonym: CDK11), CDK11B (synonym: PITSLRE), and CDK3, were mainly identified by KinOrtho alone (average overlap ratios ranged from 0.10 to 0.35). Notably, from the 78 reference proteomes, KinOrtho uniquely identified six CDK19, three CDK3, and one CDK17 ortholog. To validate these ten KinOrtho-unique CDK orthologs, we built a CDK family tree using these ten sequences and the CDK orthologs identified by at least 90% of the compared methods (Figure S4). We found that all these ten were clustered in correct subfamilies. Out of the six KinOrtho-unique CDK19 orthologs, two were the outgroup of CDK8 orthologs, and four were the outgroup of both CDK8 and CDK19 orthologs within the CDK8 subfamily. The CDK17 ortholog uniquely identified by KinOrtho was the outgroup of all CDK16, CDK17, and CDK18 orthologs within the PCTAIRE subfamily. Although two of the KinOrtho-unique CDK3 orthologs were called "CDK1" in *C.albicans* and Baker's yeast, the phylogenetic tree showed more closer either CDK3 or CDK2 instead of CDK1. The phylogenetic analysis provides additional support for these KinOrtho-unique orthologs. Although other orthology inference methods also identify these orthologs as orthologous groups, experimental characterization of these subfamilies will be needed to establish their true orthology.

KinOrtho delineates orthologs based on the protein kinase domain against orthologs based on other conserved domains

Unlike most traditional orthology detection methods, KinOrtho combines orthologs based on full-length sequences with orthologs found using only the kinase domains. It permits KinOrtho to distinguish orthologs at the protein domain level, allowing for the identification and selection of orthologs that share a closer functional relationship based on the domain of interest, here the kinase domain. On the other hand, this utility also helps identify orthologs stemming from regions other than the kinase domain, potentially sharing no kinase function similarity and leading to erroneous orthologs. Figure S6a illustrates an example of such a relationship. Instead of the kinase domain, other functional domains defined by Pfam [1] are getting matched for inferring orthology for Peripheral plasma membrane protein CASK (CASK). Nearly 20% of human kinase orthologs were inferred based on similarity in non-kinase domains (Figure S6b). KinOrtho effectively flagged such relationships, thus preventing misleading inferences of function and regulation. Within the human kinome, we found a higher ratio for orthologs missing the kinase domain in the calcium and calmodulin-regulated kinase (CAMK) group (6 out of the top 10 kinases; Figure S6c). This could be attributed to the long length of the protein sequence and the number of functional domains. The sequence lengths of four gigantic CAMK kinases, Kalirin (KALRN; synonym: Trad), Triple functional domain protein (TRIO), Obscurin (OBSCN), and Titin (TTN), range from 2,986 to 34,350 amino acids with 12, 13, 57, and 310 functional domains, respectively. On average, the human protein kinases with more than 750 amino acids have a higher ratio of full-length orthologs missing the kinase domain than the shorter kinases have (ratio = 0.043 vs. 0.004; p-value = 3.0e-8, Wilcoxon Rank Sum Test).

*Correspondence: nkannan@uga.edu
[1] Institute of Bioinformatics, University of Georgia, 120 Green St., 30602, Athens, GA, USA
[3] Department of Biochemistry and Molecular Biology, University of Georgia, 120 Green St., 30602, Athens, GA, USA
Full list of author information is available at the end of the article

Besides, the human protein kinases with more than one functional domain have a higher ratio than those with one functional domain have (ratio = 0.036 vs. 0.002; p-value = 2.9e-11, Wilcoxon Rank Sum Test). For such sequences with multiple domains, KinOrtho offers the flexibility to identify orthologs based on the domain of interest.

Comparison with KinBase, a database with domain-based kinase classifications

We next evaluated the congruence of KinOrtho with the current widely used classification of the human kinome developed by Manning et al. [2]. We retrieved the Manning classification of kinomes for humans and 14 other model organisms from KinBase [3]. We first applied KinOrtho to these 15 model organisms' proteomes to collect kinase orthologous relationships (Table S3). We identified 9,282 orthologous relationships, 4,210 of which were ortholog pairs. Because KinBase has no orthology information, if an ortholog pair identified by KinOrtho has two protein members classified as the same family/subfamily by KinBase, we consider this ortholog pair is in agreement with KinBase classification. Among the 4,210 ortholog pairs, there were 3,658 (86.9%) pairs between two proteins of the same classification. After investigating the disagreement between KinOrtho and KinBase, we found that ABC1, Alpha, PIKK, and RIO families in KinBase are classified into multiple groups. For example, a human understudied kinase "Uncharacterized aarF domain-containing protein kinase 2" (ADCK2) is classified as the ABC1-C subfamily of the ABC1 family under the Atypical group. However, its ortholog in L.major is classified as the same subfamily and family under the protein kinase-like (PKL) group. After correction for these four families, there were 3,777 (89.7%) ortholog pairs in agreement with KinBase classification. In the remaining 433 ortholog pairs, 346 had at least one protein belonging to either a species-specific or unclassified family/subfamily (named with "-Unique" or "-Unclassified"); all the others had two proteins classified as the same family but different subfamilies, except for two ortholog pairs. These two pairs were Tyrosine-protein kinase receptor UFO (AXL) proteins in humans and mice vs. their ortholog in *A.queenslandica*, "AqueK368". In KinBase, the AXL kinase domain is classified as the AXL family under the TK group, while AqueK368 is classified as the MET family under the TK group. Based on the profile hidden Markov models (HMM) analysis performed by KinBase using its in-house profile, AqueK368 can be classified as either the AXL or MET family with close scores.

KinOrtho is also a profile- and annotation-free orthology inference method

In addition to KinOrtho's query-centric characteristic, it is also a profile- and annotation-free tool that identifies target sequences' functional domains based solely on BLAST's result, unlike other domain-based methods requiring prerequisite domain annotations [4, 5, 6, 7, 8]. To check if adding Pfam's detailed annotation leads to improved orthology detection, we collected 22 thousand protein sequences with Pfam annotated protein kinase domains ("Pkinase" or "Pkinase_Tyr" in Pfam) as query sequences (Table S4). Using these expanded query sequences required 30 times sequence comparisons during the initial homology search and the following all-vs-all homology search. At the expense of computation, the number of orthologs only increased by 9.8%, and the performance was not significantly better or even worse in some metrics ("Pfam" and "PfamSub" in Figure S9), supporting the simplified BLAST-based domain searching implemented in KinOrtho.

Intrinsic issue of in-paralogs identification

In the experiment of using expanded query sequences based on Pfam annotations, we found that the ratio of in-paralogs over all orthologous relationships increased from 2.5% to 4.8% (Table S4). Moreover, when we applied KinOrtho to the proteomes in KinBase, the ratio of in-paralogs significantly increased to 15.6% (Table S3). It is an intrinsic issue of finding real in-paralogs. Under a limited number of species, many proteins cannot find a close sequence from other species, so that they might be defined as the in-paralogs of some proteins in their species. As a graph-based method, KinOrtho also takes in-paralogs into account when identifying orthologous groups. Although the cluster analysis helped KinOrtho ease this issue, an extensive collection of reference proteomes across the entire tree of life is needed to infer orthologous relationships using graph-based methods accurately.

Author details
[1] Institute of Bioinformatics, University of Georgia, 120 Green St., 30602, Athens, GA, USA. [2] PREP@UGA, University of Georgia, 500 D.W. Brooks Drive, 30602, Athens, GA, USA. [3] Department of Biochemistry and Molecular Biology, University of Georgia, 120 Green St., 30602, Athens, GA, USA. [4] Department of Biochemistry and Systems Biology, University of Liverpool, Crown St, Liverpool, UK.

References
1. El-Gebali, S., Mistry, J., Bateman, A., Eddy, S.R., Luciani, A., Potter, S.C., Qureshi, M., Richardson, L.J., Salazar, G.A., Smart, A., Sonnhammer, E.L.L., Hirsh, L., Paladin, L., Piovesan, D., Tosatto, S.C.E., Finn, R.D.: The Pfam protein families database in 2019. Nucleic Acids Res **47**(D1), 427–432 (2019)
2. Manning, G., Whyte, D.B., Martinez, R., Hunter, T., Sudarsanam, S.: The protein kinase complement of the human genome. Science **298**(5600), 1912–1934 (2002)

3. Manning, G.: KinBase: Genomics, evolution and function of protein kinases (2002)
4. Storm, C.E., Sonnhammer, E.L.: Comprehensive analysis of orthologous protein domains using the HOPS database. Genome Res **13**(10), 2353–2362 (2003)
5. Krishnamurthy, N., Brown, D., Sjölander, K.: FlowerPower: clustering proteins into domain architecture classes for phylogenomic inference of protein function. BMC Evol Biol **7** **Suppl 1**, 12 (2007)
6. Chen, T.W., Wu, T.H., Ng, W.V., Lin, W.C.: DODO: an efficient orthologous genes assignment tool based on domain architectures. Domain based ortholog detection. BMC Bioinformatics **11** **Suppl 7**, 6 (2010)
7. Uchiyama, I., Mihara, M., Nishide, H., Chiba, H.: MBGD update 2015: microbial genome database for flexible ortholog analysis utilizing a diverse set of genomic data. Nucleic Acids Res **43**(Database issue), 270–276 (2015)
8. Persson, E., Kaduk, M., Forslund, S.K., Sonnhammer, E.L.L.: Domainoid: domain-oriented orthology inference. BMC Bioinformatics **20**(1), 523 (2019)
9. Metz, K.S., Deoudes, E.M., Berginski, M.E., Jimenez-Ruiz, I., Aksoy, B.A., Hammerbacher, J., Gomez, S.M., Phanstiel, D.H.: Coral: Clear and Customizable Visualization of Human Kinome Data. Cell Syst **7**(3), 347–350 (2018)

Table S1 Statistics of KinOrtho analysis on the UniProt reference proteomes

**Dataset**			**UniProt v2019_11**	
**Method**			**Full-length**	**Domain-based**
**Query sequences**			545	558
**Reference Proteomes**	Eukaryota	Species	1202	
		Protein	18870318	
	Bacteria	Species	6554	
		Protein	25095701	
	Archaea	Species	285	
		Protein	663431	
	Viruses	Species	9093	
		Protein	468953	
	Total	Species	17134	
		Protein	45098403	
**Homology Search Result**	Eukaryota	Species	1202	1202
		Protein	792208	457181
	Bacteria	Species	6547	6302
		Protein	150425	65004
	Archaea	Species	275	260
		Protein	3174	1439
	Viruses	Species	511	342
		Protein	1952	911
	Total	Species	8535	8106
		Protein	947759	524535
**Orthology Inference Result**	Ortholog	Query*	198910	192883
		All	285080805	137330826
	In-Paralog	Query*	1	0
		All	547418	564802
	Co-Ortholog	Query*	9287	8173
		All	16248151	10351540
	Total	Query*	208198	201056
		All	301876374	148247168
**Cluster Analysis Result**	Ortholog	Query*	162005	166970
		All	93021218	97225616
	In-Paralog	Query*	1	0
		All	93566	95547
	Co-Ortholog	Query*	5043	3742
		All	3535093	2896245
	Total	Query*	167049	170712
		All	96649877	100217408
**Overlapping Orthologous Relationships**	Ortholog	Query*	131359	
		All	74063094	
	In-Paralog	Query*	0	
		All	30949	
	Co-Ortholog	Query*	1602	
		All	1348625	
	Total	Query*	132961	
		All	75442668	

Query*: the relationship is between a query sequence and any other sequences.

Table S2 GO annotation prediction results in test sets

		**Predicted**					
		**Biological process**		**Cellular component**		**Molecular function**	
		**Present**	**Absent**	**Present**	**Absent**	**Present**	**Absent**
**Existing annotation**	**Present**	6111	485	1117	33	1705	59
	**Absent**	2061	2846	345	315	234	509

The confusion matrix of each GO domain represents the number of kinase-GO term pairs observed/predicted as present/absent.

Table S3 Statistics of KinOrtho analysis on KinBase

**Dataset**			**KinBase**	
**Method**			**Full-length**	**Domain-based**
**Query sequences**			545	558
**Reference Proteomes**	Eukaryota	Species	15	
		Protein	7597	
**Homology Search Result**	Eukaryota	Species	15	15
		Protein	5749	5680
**Orthology Inference Result**	Ortholog	Query*	1740	1726
		All	6740	6638
	In-Paralog	Query*	12	4
		All	4657	5385
	Co-Ortholog	Query*	1065	893
		All	10375	10376
	Total	Query*	2817	2623
		All	21772	22399
**Cluster Analysis Result**	Ortholog	Query*	1515	1525
		All	5344	5293
	In-Paralog	Query*	12	4
		All	3286	4603
	Co-Ortholog	Query*	772	724
		All	5981	5835
	Total	Query*	2299	2253
		All	14611	15731
**Overlapping Orthologous Relationships**	Ortholog	Query*	1281	
		All	4210	
	In-Paralog	Query*	12	
		All	1452	
	Co-Ortholog	Query*	506	
		All	3620	
	Total	Query*	1799	
		All	9282	
**Consistent with KinBase Classification**	Ortholog	Query*	1164	
		All	3658	
	In-Paralog	Query*	1	
		All	289	
	Co-Ortholog	Query*	2	
		All	206	
	Total	Query*	1167	
		All	4153	

Query*: the relationship is between a query sequence and any other sequences.

Table S4 Statistics of KinOrtho analysis on QfO 2018

**Dataset**			**QfO 2018**							
**Query seq**			**Human kinases**		**Human kinases**		**Human Pkinase(_Tyr)**		**All Pkinase(_Tyr)**	
**Method**			**Full-length**	**Domain-based**	**Full-length***	**Domain-based***	**Full-length**	**Domain-based**	**Full-length**	**Domain-based**
**#Query seq**			545	558	545	558	475	488	21825	22875
**Reference Proteomes**	Eukaryota	Species	48		48		48		48	
		Protein	885338		885338		885338		885338	
	Bacteria	Species	23		23		23		23	
		Protein	82507		82507		82507		82507	
	Archaea	Species	7		7		7		7	
		Protein	17317		17317		17317		17317	
	Total	Species	78		78		78		78	
		Protein	985162		985162		985162		985162	
**Homology Search Result**	Eukaryota	Species	48	48	48	48	48	48	48	48
		Protein	22887	19006	24179	20084	19244	16606	53738	22918
	Bacteria	Species	18	19	21	22	11	12	23	18
		Protein	202	81	258	149	39	33	1048	168
	Archaea	Species	7	7	7	7	2	0	7	7
		Protein	48	25	55	30	2	0	99	11
	Total	Species	73	74	76	77	61	60	78	73
		Protein	23137	19112	24492	20263	19285	16639	54885	23097
**Orthology Inference Result**	Ortholog	Query*	9578	9689	9625	9780	7863	7984	99958	97122
		All	148655	124361	153252	126407	107977	94720	260396	101950
	In-Paralog	Query*	1	4	1	4	1	4	10132	8178
		All	10212	6870	10693	7091	9544	6320	36990	15609
	Co-Ortholog	Query*	1334	1093	1351	1114	1148	908	43662	36875
		All	56358	41038	58516	42104	47916	34250	141923	58249
	Total	Query*	10913	10786	10977	10898	9012	8896	153752	142175
		All	215225	172269	222461	175602	165437	135290	439309	175808
**Cluster Analysis Result**	Ortholog	Query*	8817	8955	8836	9034	7219	7358	82667	81338
		All	104644	101978	104885	103579	77390	76415	111625	84735
	In-Paralog	Query*	1	4	1	4	1	4	7717	6360
		All	4732	3395	4813	3119	4154	2892	18676	11387
	Co-Ortholog	Query*	867	769	875	784	731	623	29572	25105
		All	27998	23901	28865	24258	22097	18767	57050	35424
	Total	Query*	9685	9728	9712	9822	7951	7985	119956	112803
		All	137374	129274	138563	130956	103641	98074	187351	131546
**Overlapping Orthologous Relationships**	Ortholog	Query*	7820		7839		6389		67740	
		All	85262		85716		63112		69308	
	In-Paralog	Query*	1		1		1		4148	
		All	2587		2436		2144		4265	
	Co-Ortholog	Query*	471		478		369		14332	
		All	14653		15103		11174		14464	
	Total	Query*	8292		8318		6759		86220	
		All	102502		103255		76430		88037	
**Common**	Ortholog		83869				58732			
	In-Paralog		2361				1913			
	Co-Ortholog		14296				9564			
	Total		100526				70209			

Full-length*: full-length pipeline with e-value threshold = 10^1. Domain-based*: domain-based pipeline with e-value threshold = 10^1. Query*: the relationship is between a query sequence and any other sequences. Pkinase(_Tyr): the domain "Pkinase" and "Pkinase_Tyr" in Pfam.

Table S5 Statistics of compared methods

**Dataset**	**All Orthologous Relationships**		**One-to-one Orthologs**		**One-to-one Kinase Orthologs**		**One-to-one Human Kinase Orthologs**	
	**#Proteins**	**#Pairs**	**#Proteins**	**#Pairs**	**#Proteins**	**#Pairs**	**#Proteins**	**#Pairs**
BBH	637164	5675032	616400	4982139	**15091**	**117139**	9390	8848
Broccoli_1.1	715476	17222075	458320	3460218	**11587**	**74127**	7071	6560
eggNOG	634525	7813959	461494	3349588	**11335**	**75355**	7431	6898
Ensembl_Compara	675806	34069421	445997	3276281	**11782**	**77550**	7545	7015
Hieranoid 2	637280	9644226	444732	3134237	**10712**	**70091**	7233	6694
InParanoid	690553	12568693	474111	3347085	**11719**	**73720**	7345	6806
OMA_GETHOGs_2.0	588977	132246932*	399840	1766151	**9549**	**40975**	5997	5463
OMA_Groups_2.0	550127	3404204*	550127	3404204	**13344**	**79556**	8454	7910
OMA_Pairs_2.0	623745	8844020	504571	2899517	**14603**	**70750**	7477	6938
OrthoFinder_2_BLAST_DendroBLAST	772661	14990262	476054	3552212	**11512**	**78116**	7459	6922
OrthoFinder_2_BLAST_MSA	772314	14108708	468993	3477221	**11995**	**76205**	7325	6791
OrthoFinder_2_Defaults_DIAMOND_MSA	708609	12180481	462031	3452787	**11362**	**77090**	7414	6880
OrthoFinder_2_DIAMOND_MSA	708604	13040690	453227	3379401	**11402**	**76267**	7348	6819
OrthoInspector_3.0	666028	10848200	516557	3766843	**11929**	**79729**	7511	6972
PANTHER_14.1_all	633734	16419904	385486	2991733	**10078**	**63146**	6558	6030
PANTHER_14.1_LDO_only	478653	4824919	458290	4425386	**11973**	**111527**	9454	8919
Proteinortho_6.0.13_with-isoform	554665	3523573	492492	2590594	**11857**	**63170**	7057	6532
RSD	716963	5770391	697797	5123209	**19752**	**120871**	9469	8925
SonicParanoid_default	659776	13298118	463644	3219094	**11406**	**81277**	7573	7034
SonicParanoid_most	690730	14579191	480923	3699995	**11552**	**82829**	7618	7079
SonicParanoid_sensitive	683736	14189929	477422	3594286	**11516**	**82667**	7613	7074

*: consider all members' combinations in an orthologous group. Statistics of the datasets we submitted to Ortholog Benchmarking Webservice are highlighted in bold.

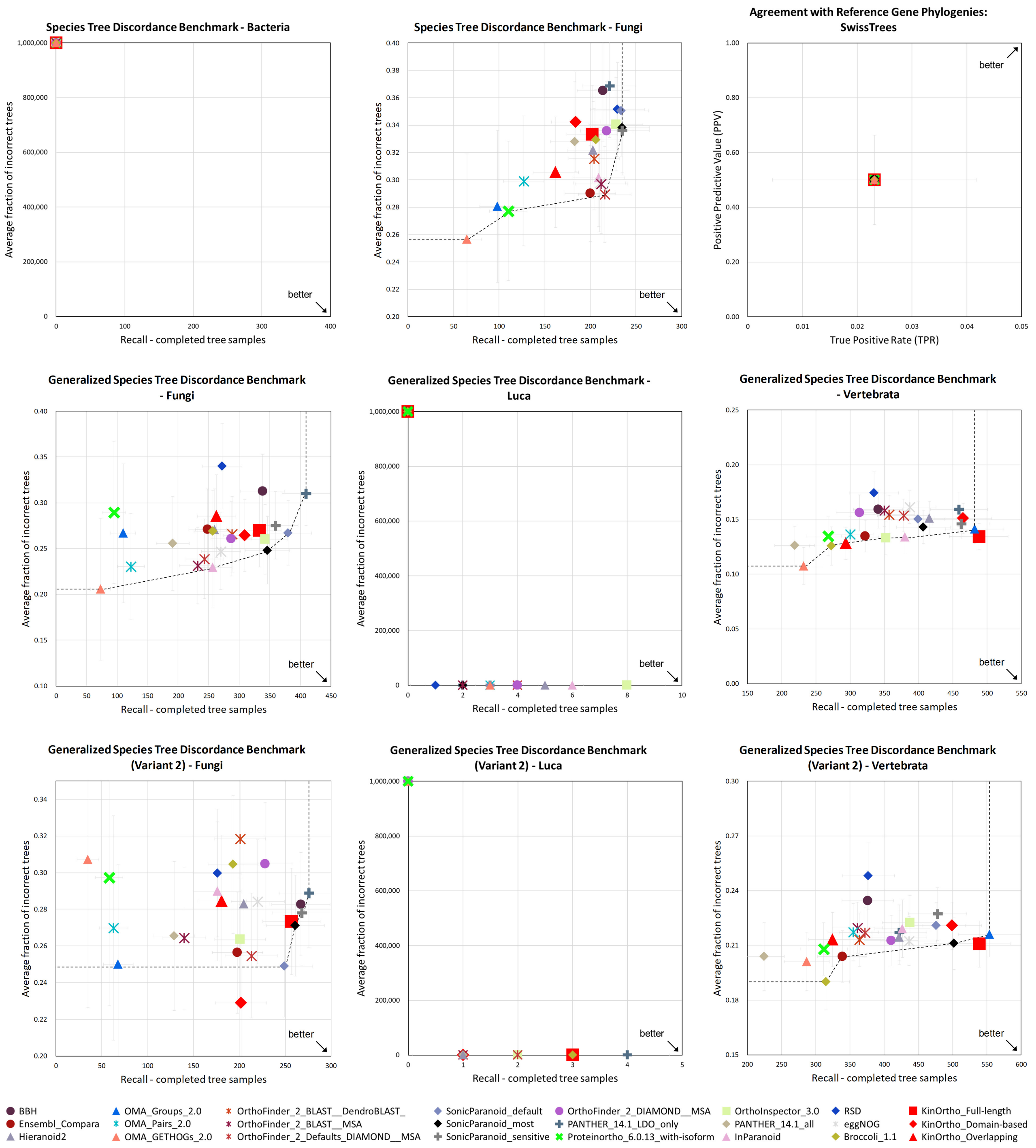

Figure S1 Benchmarking based on nine metrics. The title of each plot represents the evaluation metric. KinOrtho results are marked in red. The dotted line represents the Pareto frontier, which runs over the participants with the best efficiency (except KinOrtho). The arrow in the plot shows the optimal corner.

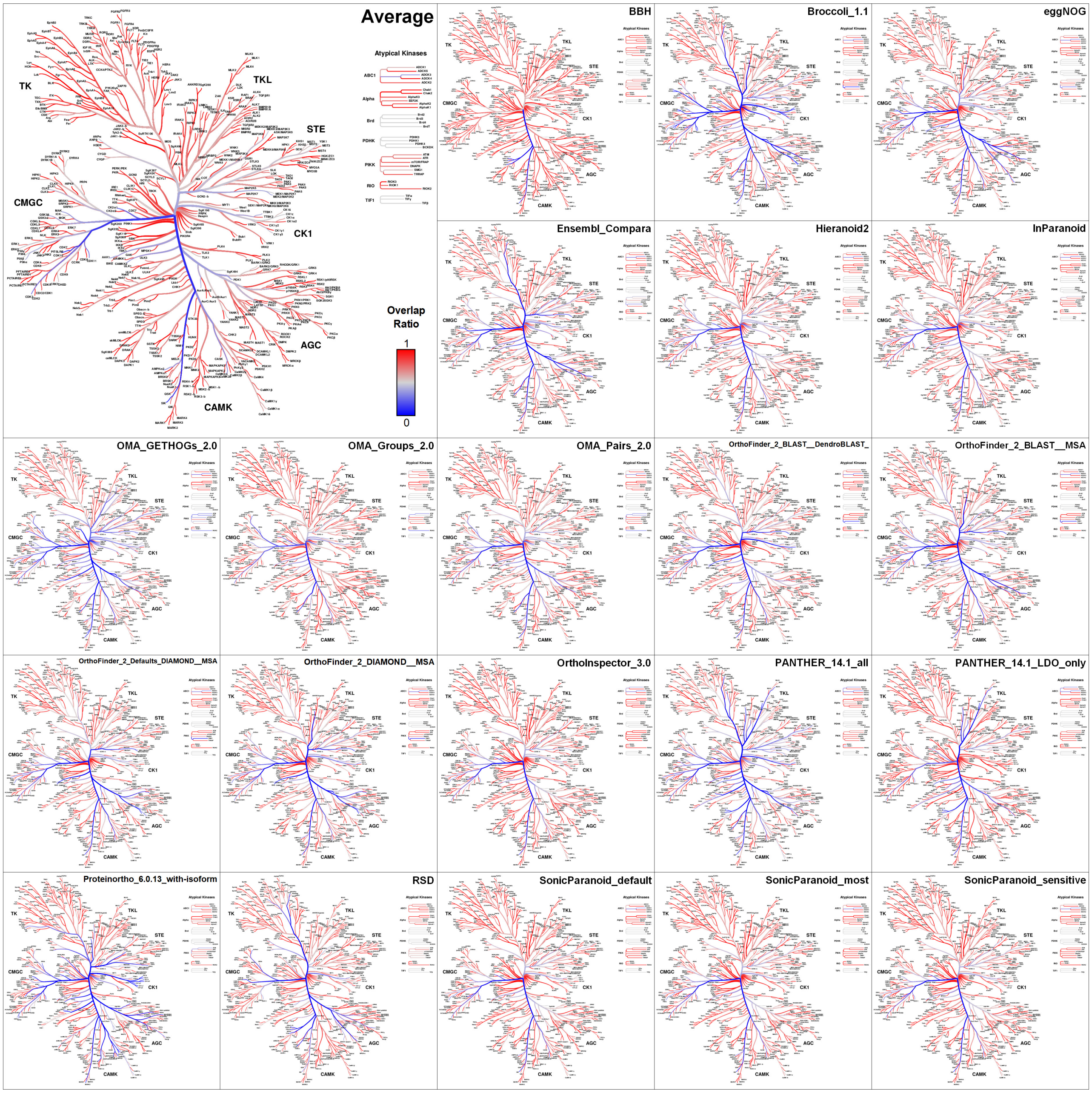

Figure S2 Comparison between KinOrtho with other methods in identifying the orthologs of human kinases. Overlap ratio is defined as the overlap of orthologs identified by KinOrtho and other methods divided by the number of orthologs identified by KinOrtho. The human kinome tree was generated using Coral [9]. The illustration is reproduced courtesy of Cell Signaling Technology, Inc. (http://www.cellsignal.com).

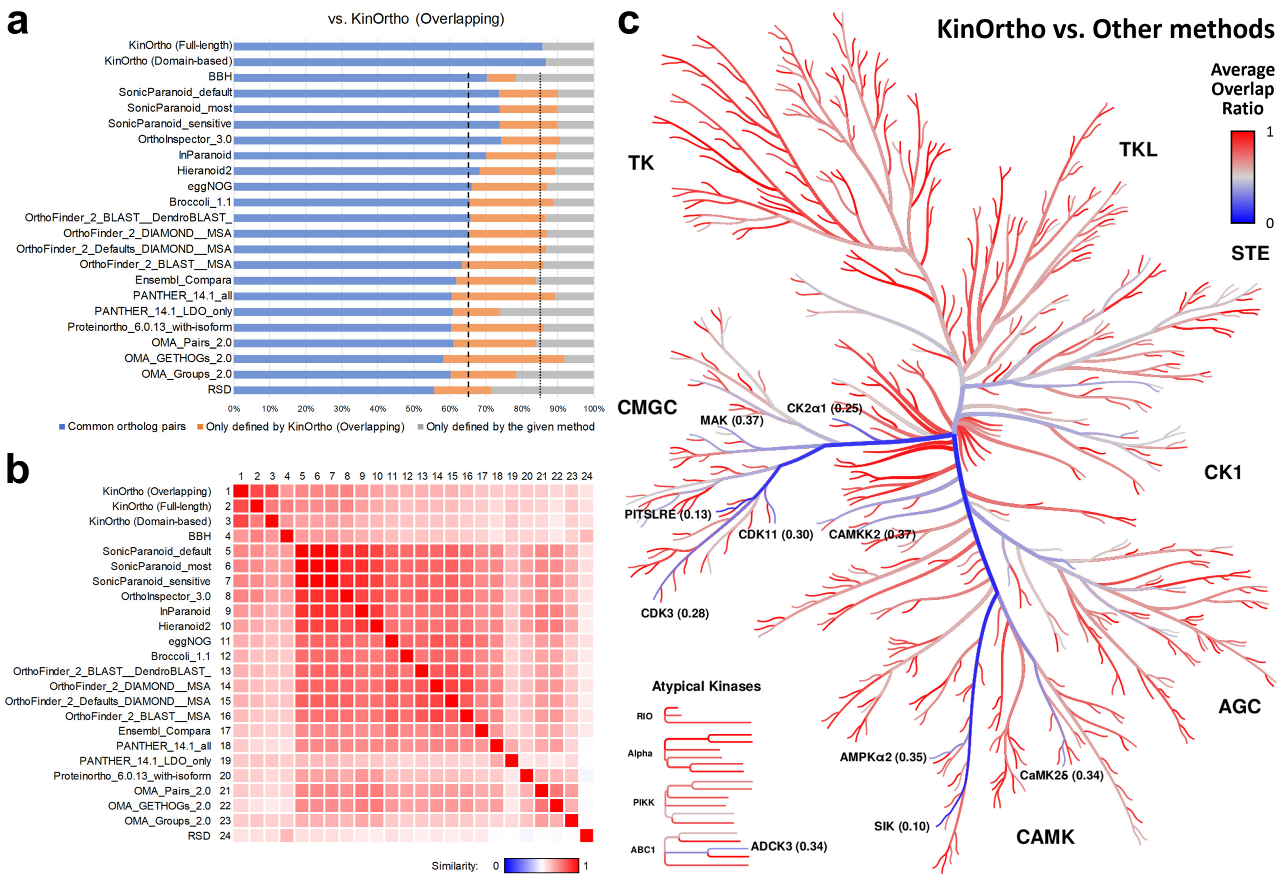

Figure S3 Comparison with other methods in identifying the orthologs of human kinases. (a) The 100% stacked bar chart shows the overlap in identifying human kinase orthologs using KinOrtho versus other methods (blue region); a dashed line indicates the average percentage of the overlaps (KinOrtho Full-length and KinOrtho Domain-based do not count). The orange region represents the percentage of orthologs only identified by KinOrtho; a dotted line indicates the average percentage of the blue and orange regions. The gray region shows the percentage of orthologs unique to the compared method. (b) The heat map represents the pairwise similarity (measured by the Jaccard index) between orthology inference methods in identifying human kinase orthologs. (c) Visualization of the average overlap ratio between KinOrtho and other methods in the human kinome tree. The ratio is defined as the average overlap of orthologs identified by KinOrtho and other methods divided by the number of orthologs identified by KinOrtho. The kinases with the lowest 10 ratios are labeled. The illustration is reproduced courtesy of Cell Signaling Technology, Inc. (http://www.cellsignal.com).

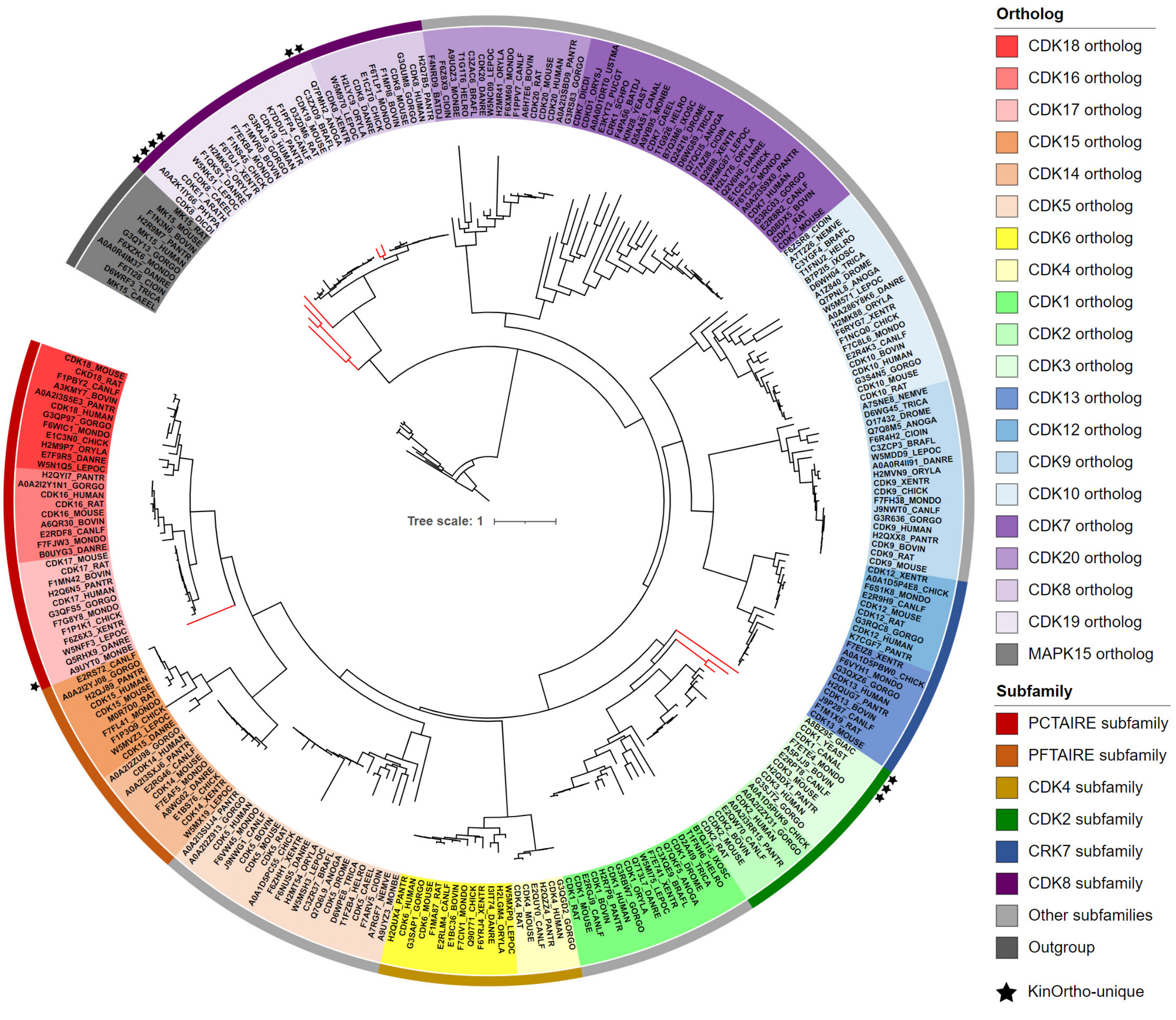

Figure S4 CDK family tree. The orthologs of CDK19, CDK17, or CDK3 uniquely identified by KinOrtho (overlapping set) are highlighted in red branches and indicated by black stars.

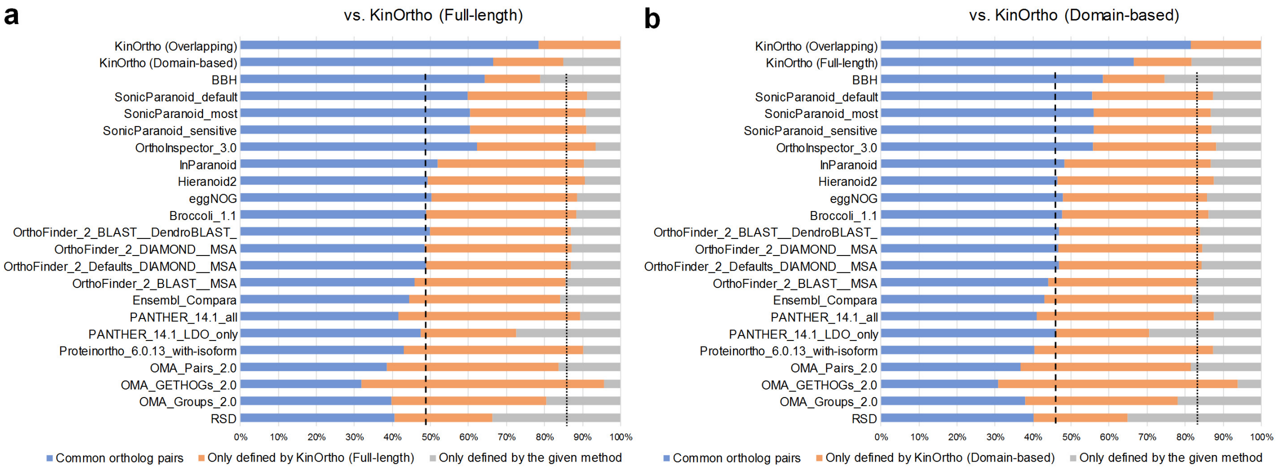

Figure S5 Comparison between KinOrtho with other methods. (a) KinOrtho (Full-length) vs. other methods. A dashed line indicates the average percentage of the overlaps; a dotted line indicates the average percentage of the blue and orange regions. (b) KinOrtho (Domain-based) vs. other methods.

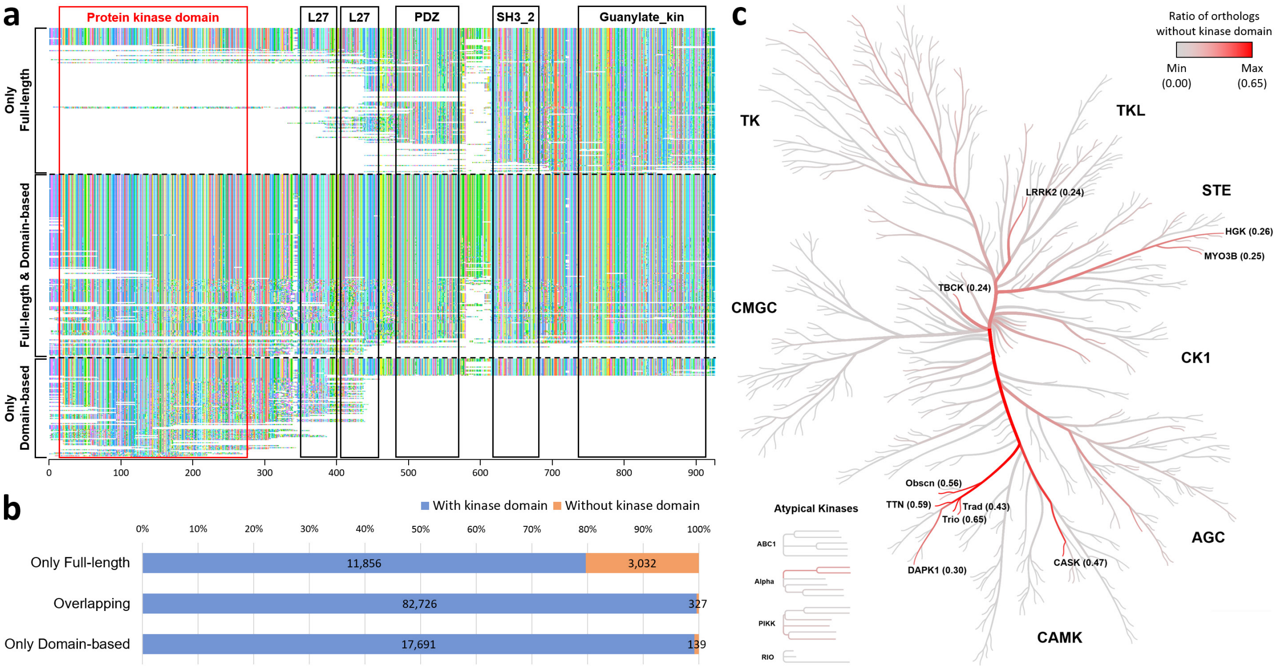

Figure S6 Comparison of known kinase domain annotations and the results of full-length and domain-based orthology inference methods. (a) Example of homology search results matching other functional domains instead of protein kinase domains. Each row represents the residues of a CASK ortholog matching human CASK by BLAST search. The labels on the left show the orthologs are identified based on the full-length, domain-based, or both methods. The residue numbering of human CASK is given at the bottom of the alignment. The boxes show the boundaries of functional domains annotated by Pfam; the protein kinase domain is highlighted in red. (b) The 100% stacked bar chart shows the percentages of orthologs with or without known protein kinase domain (shown in blue or orange, respectively) using different orthology inference methods. (c) Visualization of the ratio of orthologs without kinase domains in the human kinome tree. Kinase orthologs are identified by the full-length method. The kinases with the top 10 ratios are labeled. The illustration is reproduced courtesy of Cell Signaling Technology, Inc. (http://www.cellsignal.com).

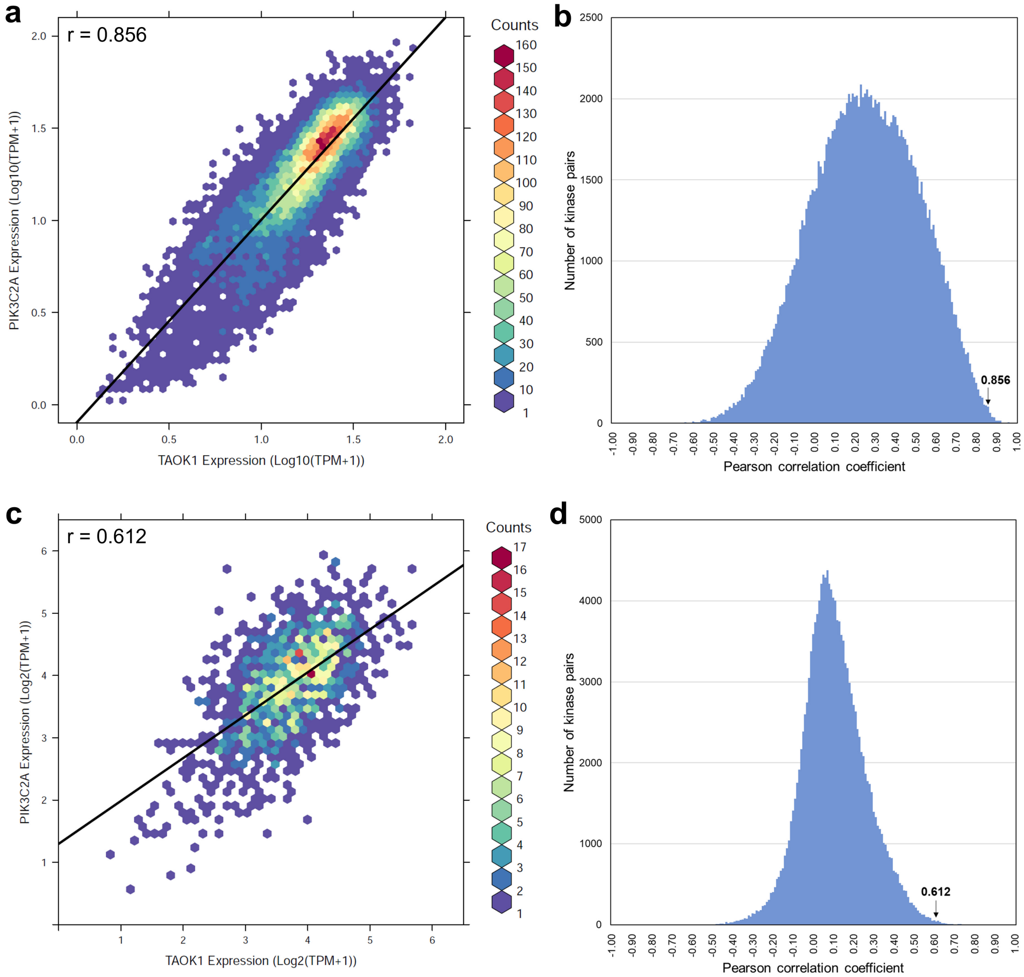

Figure S7 Co-expression analysis of human kinases. (a) Co-expression between TAOK1 and PIK3C2A in normal samples. (b) Distribution of co-expression (measured by Pearson correlation coefficient) between all pairs of human kinases in normal samples. (c) Co-expression between TAOK1 and PIK3C2A in cancer samples. (d) Distribution of co-expression between all pairs of human kinases in cancer samples.

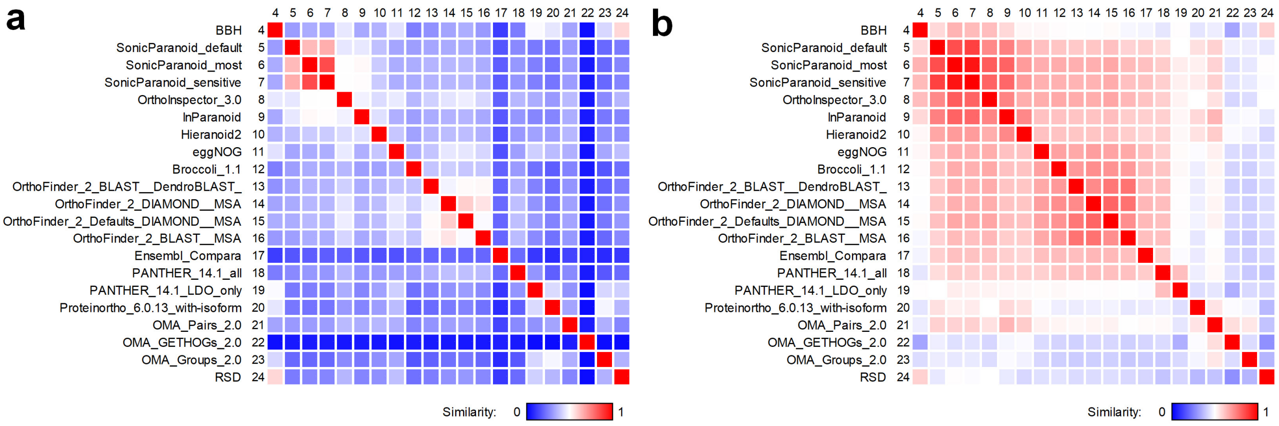

Figure S8 Jaccard similarity matrices among compared methods. Method indices (according to the order in Figure 2c) are shown on the matrices' left and top. (a) The heatmap represents the similarity among all orthologous relationships of compared methods. Average similarity = 29.9%. (b) The heatmap represents the similarity among the one-to-one orthologs of compared methods. Average similarity = 56.1%.

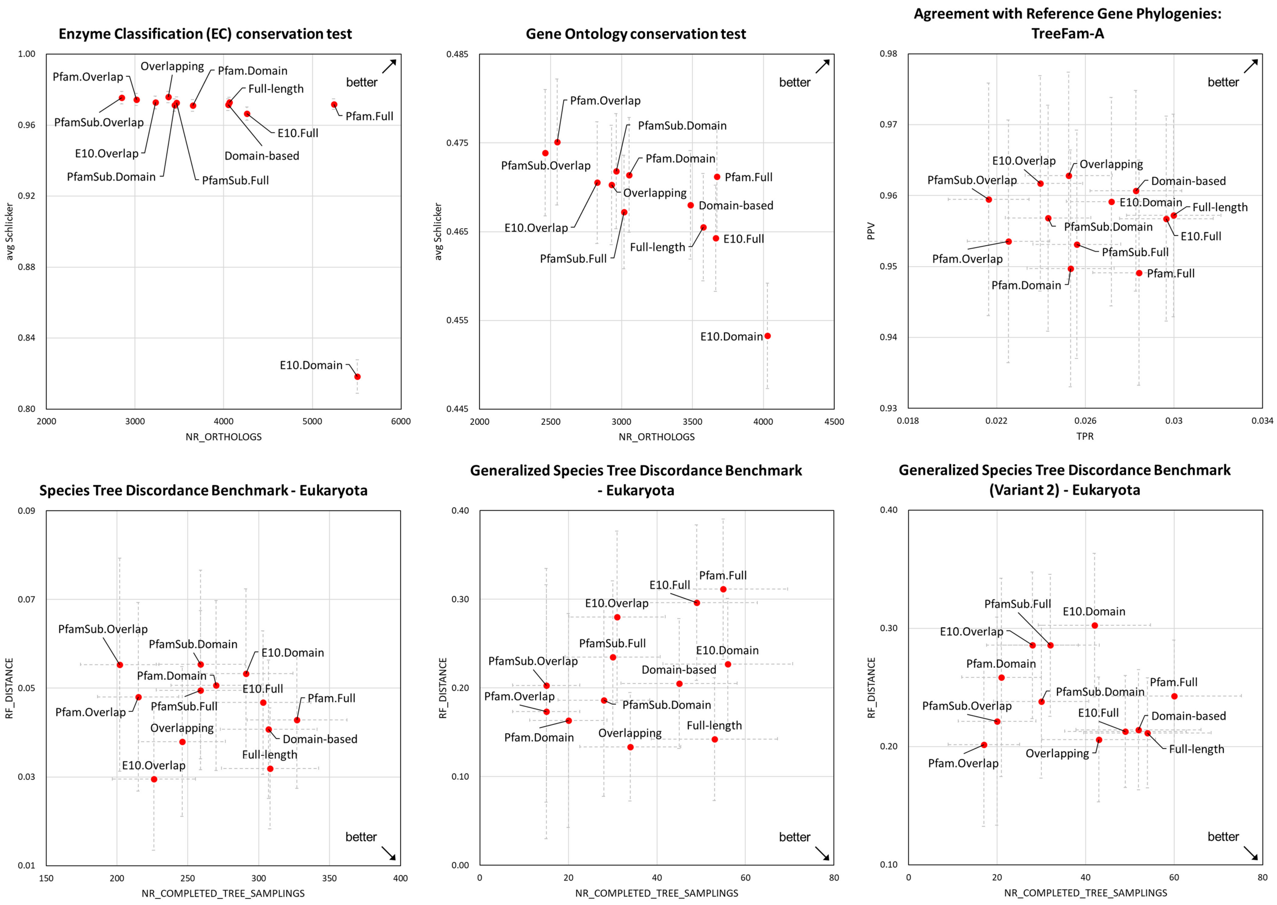

Figure S9 Evaluations of the kinase orthologs identified by KinOrtho based on different query sequences and default values. Full-length: full-length method and 545 human protein kinases as query sequences; Domain-based: domain-based method and 558 human protein kinase domains as query sequences; Overlapping: overlaps of Full-length and Domain-based results; E10.Full: full-length method with e-value threshold = 10; E10.Domain: domain-based method with e-value threshold = 10; E10.Overlap: overlaps of E10.Full and E10.Domain; PfamSub.Full: full-length method and 475 Pfam-defined human protein kinases as query sequences; PfamSub.Domain: domain-based method and 488 Pfam-defined human kinase domains as query sequences; PfamSub.Overlap: overlaps of PfamSub.Full and PfamSub.Domain; Pfam.Full: full-length method and 21,825 Pfam-defined protein kinases as query sequences; Pfam.Domain: domain-based method and 22,875 Pfam-defined kinase domains as query sequences; Pfam.Overlap: overlaps of Pfam.Full and Pfam.Domain.

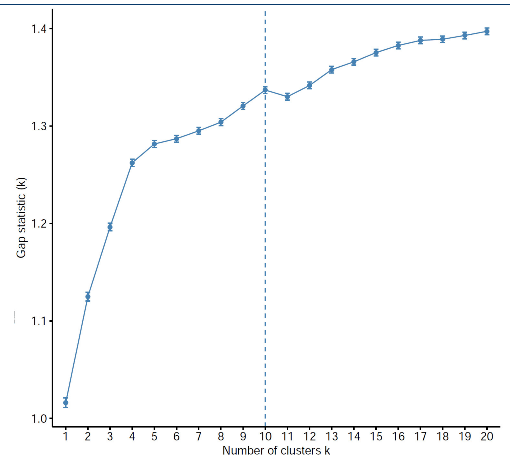

Figure S10 Determining the optimal number of clusters for clustering phylogenetic profile. The gap statistic method was used to determine the optimal number of k-means clusters. Dashed line indicates the optimal number of clusters.
